# Pterostilbene enhances reproductive outcomes and oocyte quality in aged mice without adverse effects

**DOI:** 10.18632/aging.206287

**Published:** 2025-07-23

**Authors:** Naoki Okamoto, Yorino Sato, Yuta Kawagoe, Kazuhiro Kawamura

**Affiliations:** 1 Department of Food and Reproductive Function, Juntendo University Faculty of Medicine, Bunkyo-ku, Tokyo 113-8421, Japan; 2 Department of Obstetrics and Gynecology, Juntendo University Faculty of Medicine, Bunkyo-ku, Tokyo 113-8421, Japan

**Keywords:** pterostilbene, age-associated infertility, oocyte quality, implantation, mitochondria

## Abstract

Aging is associated with a decline in oocyte quality, which reduces the likelihood of successful pregnancy and childbirth. Recent research has focused on dietary supplements to improve oocyte quality, with particular interest in resveratrol, a natural polyphenol. Pterostilbene, a dimethoxylated analog of resveratrol, has demonstrated greater physiological activity. This study investigates the effects of pterostilbene on reproductive outcomes in mice. Short-term (1-week) ingestion of pterostilbene by aged mice resulted in an increased implantation rate. In middle-aged mice, long-term (22-week) ingestion enhanced implantation and live birth rates, increased the number of ovulated oocytes, and reduced miscarriage rates, correlating with serum pterostilbene levels. Importantly, pterostilbene ingestion did not affect the estrous cycle or body weight, and offspring showed no health or reproductive abnormalities. At the cellular level, pterostilbene treatment improved mitochondrial membrane potential and ATP levels in oocytes, though it did not alter mitochondrial DNA copy number. Unlike resveratrol, pterostilbene did not inhibit decidualization in endometrial stromal cells. These findings suggest that pterostilbene effectively restores oocyte quality in aged mice and prevents age-related oocyte deterioration in middle-aged mice without adverse effects on the animals or their progeny.

## INTRODUCTION

In recent years, there has been an exponential increase worldwide in the number of infertile patients of advanced age, primarily due to socioeconomic changes. Substantial evidence indicates that age-related deterioration of oocyte quality reduces the likelihood of successful pregnancy and childbirth [1–3]. While chromosomal abnormalities have been identified as the primary cause of the age-related decline in oocyte quality [4, 5], other factors including increased cellular [6] and DNA [7] damage resulting from age-related reactive oxygen species, as well as decreased mitochondrial copy number and ATP production [8] also contribute to the diminishing oocyte quality. These abnormalities accumulate with age, progressively compromising oocyte quality. Therefore, there is an increasing need to develop methods that not only restore but also prevent the age-related decline in oocyte quality, aiming to establish effective anti-aging therapies for infertile patients of advanced age.

Natural polyphenols are plant secondary metabolites that play a crucial role in protecting plants from various stresses [9]. These compounds have also demonstrated numerous potential benefits for human health, including anticancer properties, skincare applications, and the prevention and treatment of metabolic syndrome [10–12]. Resveratrol, a common natural polyphenol found in grapes, red wine, and nuts, possesses potent antioxidant and anti-inflammatory properties and is available as a dietary supplement [9]. Furthermore, resveratrol has been shown to activate sirtuin enzymes, which are involved in anti-aging cellular processes, and to enhance mitochondrial function [13]. A previous study on young mice treated with resveratrol for 12 months demonstrated its anti-aging activity by preventing the age-related decline in oocyte quality [14]. Our recent research demonstrated that short-term resveratrol supplementation in aged mice, whose oocyte quality had already declined, improved pregnancy and birth rates while increasing mitochondrial activity and ATP production in the oocytes [15]. Despite these promising findings, a significant challenge in the clinical application of natural polyphenols like resveratrol is their short half-life, which results in low biological activity *in vivo* [16, 17].

Pterostilbene is a natural dimethoxylated analog of resveratrol, extracted from the heartwood of *Pterocarpus marsupium*, blueberries, and grapes. Its biological half-life is considerably longer than that of resveratrol, due to its high lipophilicity [18]. Pterostilbene exhibits a variety of biological functions, including antioxidant, anti-inflammatory, and anticancer properties [19]. An *in vitro* study of the reproductive system demonstrated that mouse preimplantation embryos cultured with pterostilbene exhibited enhanced development, increased expression of cell-protective genes, and decreased pro-apoptotic gene expression [19]. Our findings, in conjunction with previous research, prompted us to investigate the anti-aging activity of pterostilbene on oocyte quality in aging mice, aiming to obtain proof of concept for future therapies targeting aging infertile women. Our study revealed that short-term (1 week) ingestion of pterostilbene had a positive effect on restoring the quality of oocytes that had already declined in quality due to aging. Furthermore, long-term (22 weeks) ingestion of pterostilbene was found to be more effective than short-term ingestion. These findings suggest a possible protective effect against the age-related deterioration of oocyte quality.

## RESULTS

### Pterostilbene ingestion did not affect estrous cycle and body weight in aged mice

**Figure 1:**
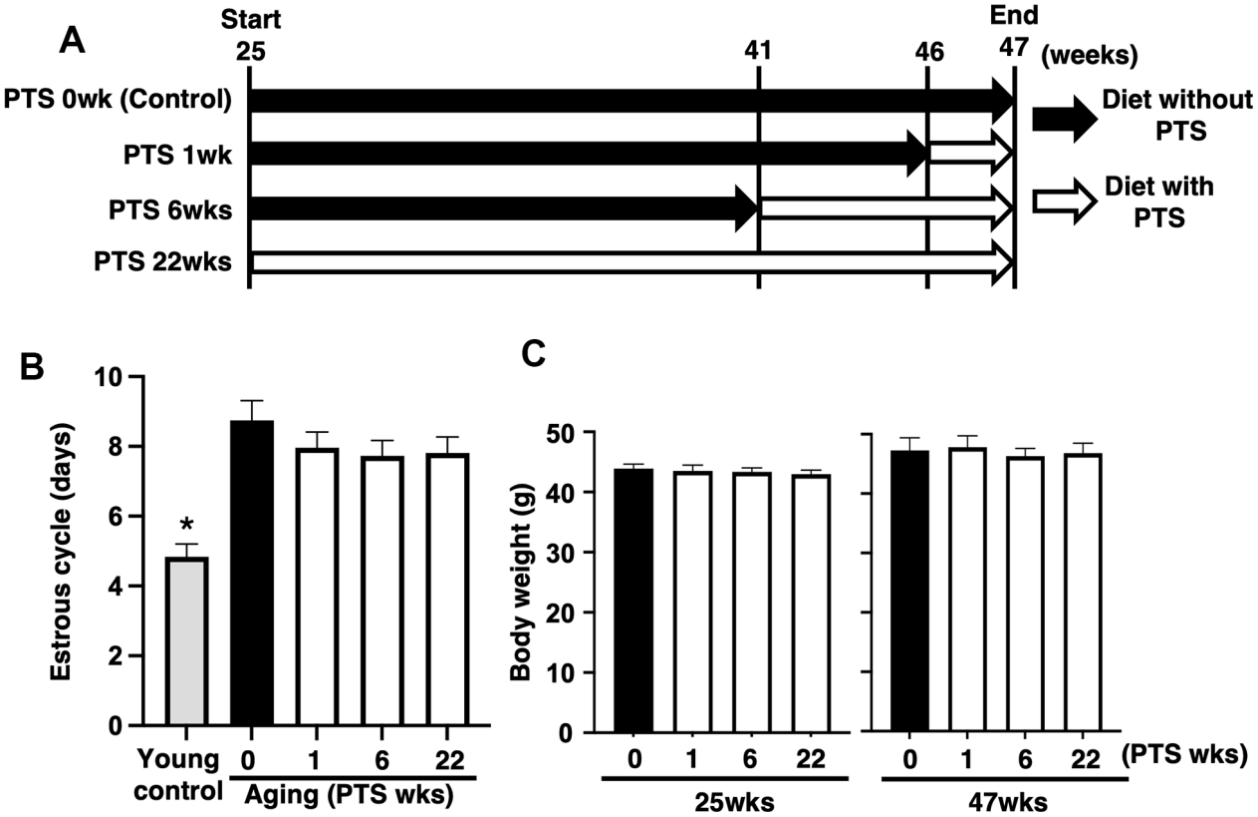
**Study design and outcomes of pterostilbene ingestion on estrous cycle and body weight during mouse aging.** (**A**) Study design for *in vivo* experiments. A total of 80 ICR mice at 25 weeks (wks) of age were housed until 47 weeks of age and fed either with or without pterostilbene (PTS). These mice were divided into four groups (20 mice in each group) based on four different feeding durations: 0 (control), 1, 6, and 22 weeks. Additionally, young mice served as controls in some experiments to confirm the effect of aging on reproduction. Mice were weighed and recorded at the start of pterostilbene ingestion (25 weeks of age) and again at 47 weeks of age. After reaching 47 weeks, ovulated oocytes were collected and counted following human chorionic gonadotropin injection for ovulation induction. Subsequently, *in vitro* fertilization-embryo transfer was performed to determine the rates of fertilization, blastocyst formation, implantation, live pups, and abortion. Some ovulated oocytes were used for analyses of mitochondrial functions. (**B**) Estrous cycles during 22 weeks of pterostilbene ingestion. Estrous cycles were evaluated by examining vaginal epithelial cell smears collected every 48 hours (n=18-20 animals, with n=78 observations per animal). For the control group, young ICR mice at six weeks of age were used as young controls. (**C**) Body weights of animals. Body weights of animals in each group were recorded at 25 and 47 weeks of age at the start and end of pterostilbene ingestion, respectively (n=18-20 animals). The bars represent the mean ± standard error (SE). *, *p* < 0.05, a significant difference between the same characters.

To evaluate the effect of pterostilbene on ovarian follicle development, changes in the estrous cycle were quantified via vaginal smears of epithelial cells. As shown in Figure 1B, the mean estrous cycle of the young control group was approximately four days, whereas that of aged mice without pterostilbene ingestion (PTS 0 week) was approximately nine days. Among the four groups of mice ingested pterostilbene for different periods (0, 1, 6, and 22 weeks), no significant difference was found in the mean pattern of the estrous cycle.

As ingestion behavior could affect follicle development through changes in body weight, we measured body weights at 25 and 47 weeks of age in groups with or without pterostilbene ingestion. Although an overall increase in body weight was observed at 47 weeks compared to 25 weeks across all groups, there was no significant difference in body weight among the four different periods of pterostilbene ingestion (Figure 1C).

### Pterostilbene ingestion improved age-related infertility in mice

To examine the anti-aging activity of pterostilbene on age-related infertility, oocytes were collected from aged mice after 47 weeks of age. The mice were divided into four groups with different durations of pterostilbene ingestion: 1) ingested with control diet (CD) throughout the breeding period (controls), 2) ingested with CD until 46 weeks of age, then ingested with pterostilbene diet (PD) for one week, 3) ingested with CD until 41 weeks of age, then ingested with PD from 6 weeks, and 4) ingested with PD throughout breeding period (Figure 1A). Oocyte quality was then evaluated using *in vitro* fertilization and embryo transfer (IVF-ET).

**Figure 2:**
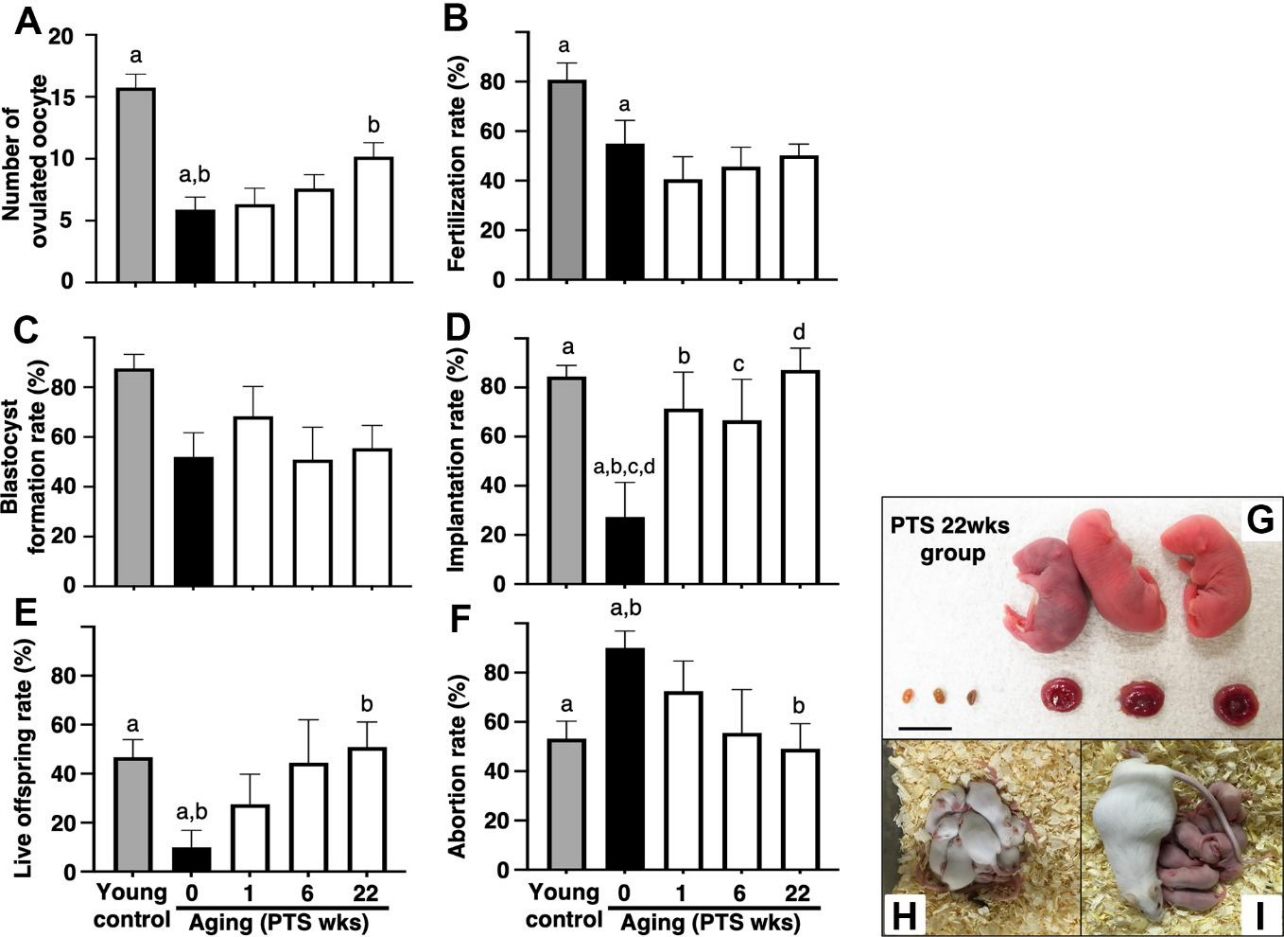
**Effects of pterostilbene ingestion on reproductive outcomes in aged mice.** Ovulation was induced at the proestruus stage following 47 weeks of pterostilbene (PTS) ingestion by administrating human chorionic gonadotropin (hCG). At 15 hours post-hCG injection, cumulus-oocyte complexes (COCs) were collected from the oviduct ampulla and subsequently inseminated with sperm from fertile male mice. At 16 hours after culture, 2-cell stage embryos were collected and allowed to develop to the blastocyst stage for an additional 72 hours of culture. Following the completion of embryo culture, the blastocysts from each animal were transferred to independent recipient mice. A Caesarian section was performed 16 days after embryo transfer to ascertain the number of implantation sites and live offspring. For the young control group, ICR mice at six weeks of age were used (young control). (**A**) Number of ovulated oocytes. The number of ovulated oocytes was quantified by the removal of cumulus cells surrounding oocytes after insemination using a stereomicroscope (n=18-20 animals). (**B**) Fertilization rate (the number of 2-cell stage embryos divided by the number of ovulated oocytes) (n=11* - 17 animals, 40-86 two-cell stage embryos per group). *, three mice in the control group, seven mice in the PTS 1-week group, four mice in the PTS 6-weeks group, and one mouse in the PTS 22-weeks group did not ovulate. (**C**) Blastocyst formation rate (the number of blastocysts divided by the number of 2-cell stage embryos) (n=10*-15 animals, 18-51 blastocysts per group). *, oocytes retrieved from three mice in the control group, one mouse in the PTS 1-week group, two mice in the PTS 6-weeks group, and one mouse in the PTS 22-weeks group did not fertilize. (**D**) Implantation rate (the number of implanted blastocysts divided by the number of transferred blastocysts) (n=9-13* animals, 10-46 implanted blastocysts per group). *, 2-cell stage embryos derived from three mice in the control group, one mouse in the PTS 1-week group, four mice in the PTS 6-weeks group, and two mice in the PTS 22-weeks group were arrested in their development before reaching the blastocyst stage. (**E**) Live offspring rate (the number of live offspring divided by the number of transferred blastocysts) (n=9-13 animals, 5-32 live offspring per group). (**F**) Abortion rate (1 minus the live offspring rate). (**G**) Representative images of live offspring and placentas derived from the PTS 22-weeks group. Scale bars, 10 mm. Following the Caesarian section, the offspring were nursed by foster mothers to assess their health status and were mated at 8 weeks of age to confirm their fertility. (**H**) The offspring at 10 days after Caesarian section. (**I**) The offspring with delivered pups. The bars represent the mean ± SE. Different letters (**A**–**D**) show significant differences (p < 0.05) between the same symbols.

The number of ovulated oocytes in the aged mice without pterostilbene ingestion (aged control) was significantly reduced compared to young counterparts (Figure 2A). Long-term pterostilbene ingestion (22 weeks) significantly increased the number of ovulated oocytes compared to the aged control group. In contrast, middle-term (six weeks: from 41 to 47 weeks of age) and short-term (one week: from 46 to 47 weeks of age) pterostilbene ingestion did not alter the number of ovulated oocytes in the aged animals (Figure 2A).

The potential of oocytes for fertilization and subsequent embryo development was assessed by examining fertilization and blastocyst formation rates. The fertilization rate decreased with aged, and pterostilbene ingestion did not restore this declined rate in aged animals (Figure 2B). In our study protocols, the blastocyst formation rate was not affected by aging; consequently, the potential recovery of blastocyst formation by pterostilbene ingestion in aged mice could not be evaluated (Figure 2C).

To further investigate the anti-aging activity of pterostilbene, embryo potential for implantation and fetal growth was evaluated by transferring blastocysts obtained from animals with or without pterostilbene ingestion to pseudo-pregnant young mice. Due to age-related deterioration in oocyte quality, implantation and live offspring rates fell below 25%, and the abortion rate doubled in aged controls compared to young counterparts (Figures 2D–2F). Pterostilbene ingestion dramatically ameliorated these outcomes. Notably, implantation, live birth, and abortion rates in the group with 22 weeks of pterostilbene ingestion reached levels comparable to those observed in young animals (Figures 2D–2F). These rates were also restored in the groups subjected to middle- and short-term pterostilbene ingestion (Figures 2D–2F).

To ensure the safety of pterostilbene administration, the gross morphology of the fetus and placenta was evaluated at Cesarean section for delivery. As shown in Figure 2G, no aberrant findings were detected in either the live fetus or the corresponding placenta derived from embryos obtained from mice treated with the longest duration of pterostilbene ingestion (22 weeks). The fetuses were subsequently nursed by foster mothers and demonstrated normal development (Figure 2H: ten days post-Cesarean section). Following the mating of the second generation of mice, their offspring exhibited normal health and development (Figure 2I).

### Serum pterostilbene levels correlated with better fertility outcomes but not Sirtuin expression in aged mice

**Figure 3:**
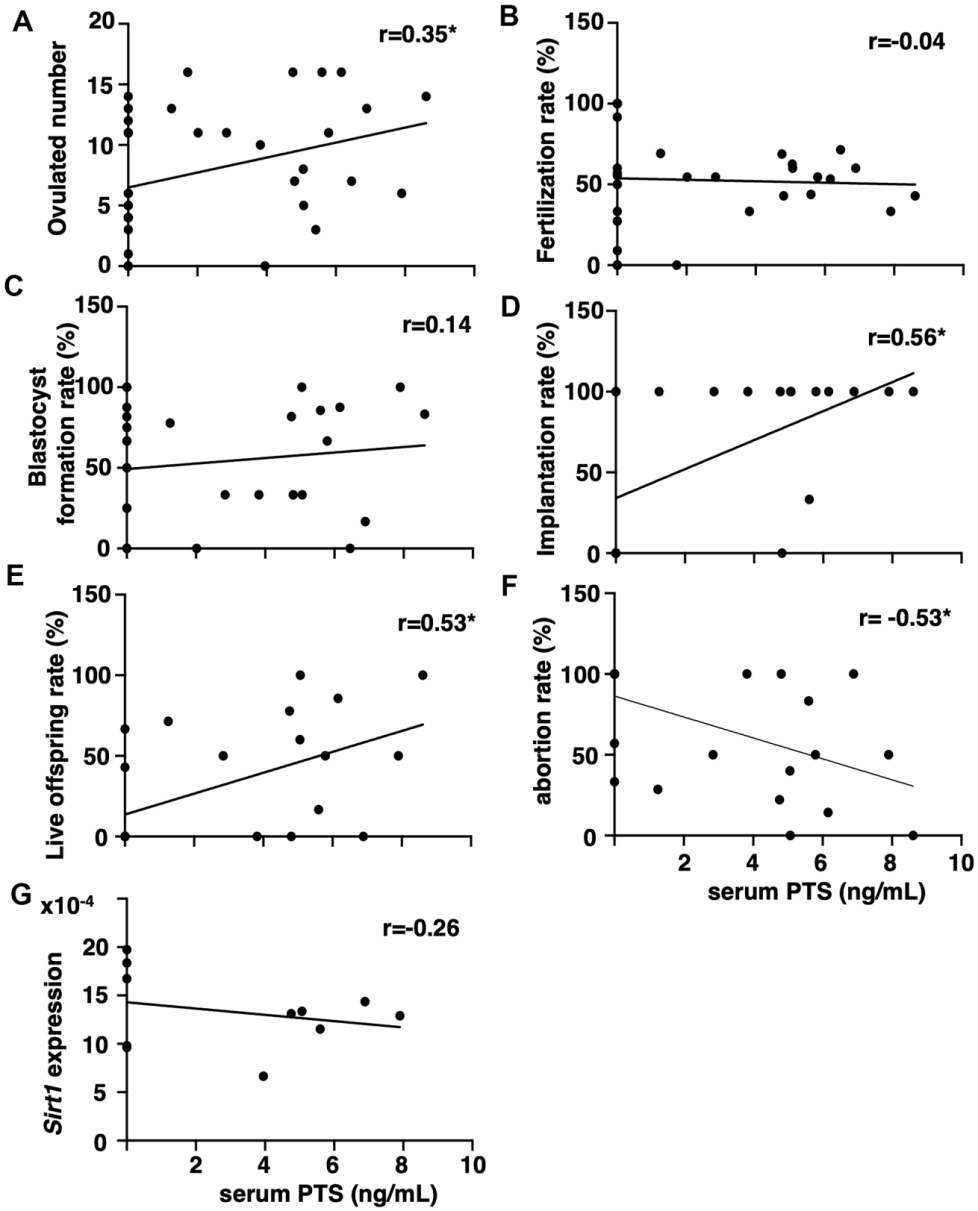
**Correlation between serum pterostilbene levels and reproductive parameters in aged mice.** Serum pterostilbene (PTS) levels were quantified in the control and 22-weeks ingestion groups using HPLC-MS/MS, while Sirt1 transcript levels were determined by real-time RT-PCR. Correlations were analyzed between serum PTS levels and: (**A**) ovulated number (n=38 animals), (**B**) fertilization rate (n=33 animals), (**C**) blastocyst formation rate (n=29 animals), (**D**) implantation rate (n=25 animals), (**E**) live offspring rate (n=25 animals), (**F**) abortion rate (n=25 animals), and (**G**) ovarian Sirt1 transcript levels (n=11 animals). A correlation coefficient (r) above ± 0.3 was considered to indicate a significant correlation.

The correlation between serum pterostilbene levels at the time of sacrifice and fertility outcomes was assessed by measuring pterostilbene levels in animals from all experimental groups using high-performance liquid chromatography (HPLC)-tandem mass spectrometry (MS/MS). After confirming the absence of pterostilbene in serum from animals without pterostilbene ingestion (aged controls), positive correlations were detected between serum pterostilbene levels and the number of ovulated oocytes, as well as implantation and live offspring rates (Figures 3A, 3D, 3E). A negative correlation was observed with the abortion rate (Figure 3F). As expected, there was no correlation between serum pterostilbene levels and fertilization or blastocyst formation rates (Figures 3C, 3D).

Because resveratrol has demonstrated anti-aging activity in age-related infertility through increased Sirtuin family expression [15], the correlation between serum pterostilbene and ovarian Sirtuin family transcript levels was further analyzed. Serum pterostilbene levels were found to be uncorrelated with the expression levels of *Sirt1* (Figure 3G), and other Sirtuin family members, *Sirt* 2-7 (Supplementary Figure 1).

### Pterostilbene ingestion maintained mitochondrial functions in oocytes from aged-mice

**Figure 4:**
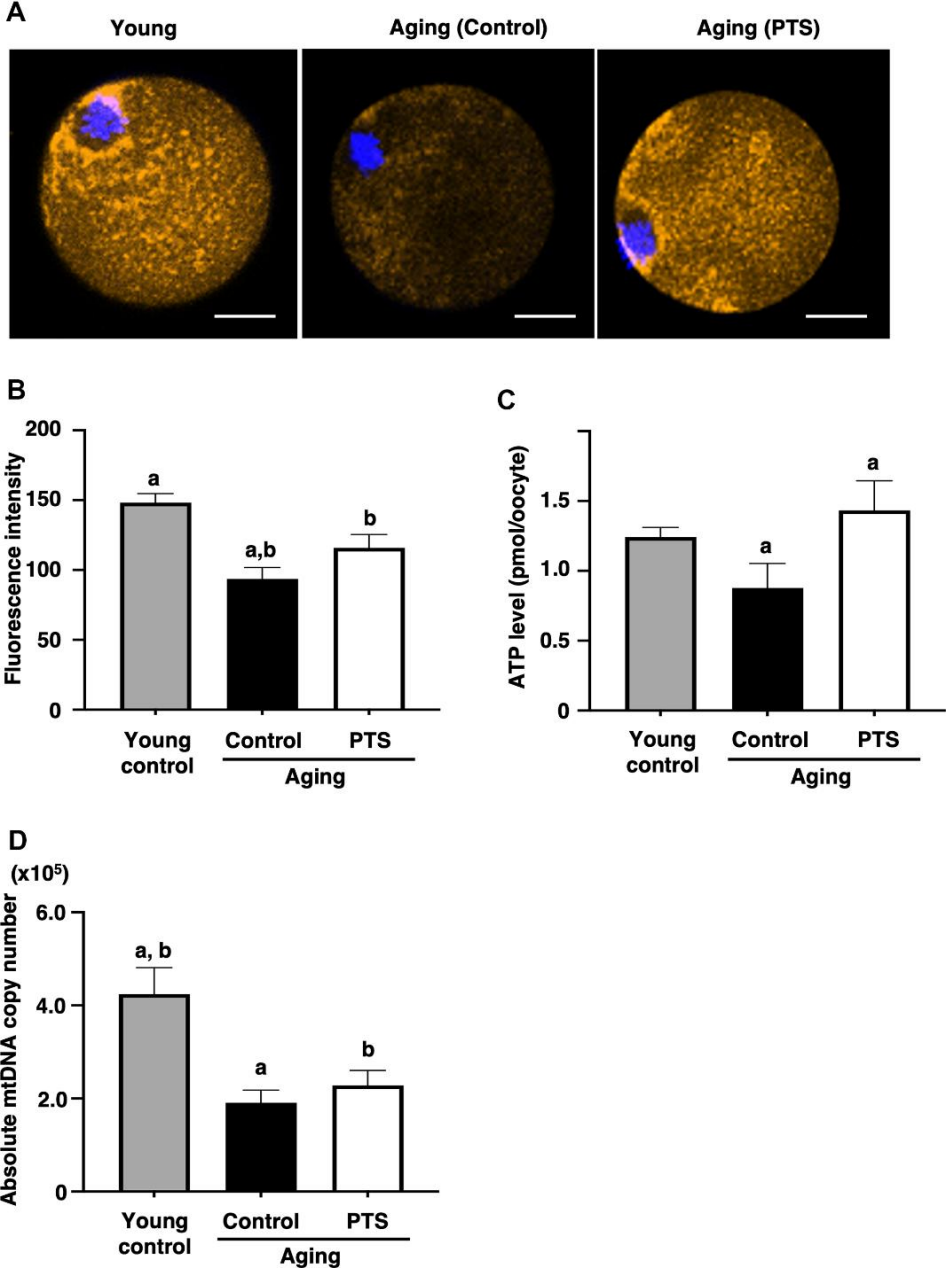
**Effects of pterostilbene ingestion on mitochondrial functions in oocytes derived from aged mice.** Mature oocytes derived from aged mice without (control) or with 22 weeks of pterostilbene (PTS) ingestion, and young animals without pterostilbene ingestion (young) were subjected to different mitochondrial assays. (**A**, **B**) Mitochondrial membrane potential. (**A**) Representative fluorescence images demonstrating mitochondrial membrane potential, visualized using the MitoTracker™ dye (orange). The oocyte nuclei were counterstained with Hoechst 33342 (blue). Scale bars, 20 μm. (**B**) The fluorescence intensities of mitochondrial membrane potential. The intensity of mitochondrial fluorescence in ooplasm of mature oocytes was measured, excluding that in the first polar body (n=10 oocytes per group). (**C**) ATP levels in mature oocytes. The ATP levels per mature oocyte were quantified using the ATP-Glo™ Bioluminometric Cell Viability Assay Kit (young: n=26, control: n=29, and PTS: n=19 oocytes). (**D**) Mitochondrial DNA (mtDNA) copy numbers of mature oocytes. The copy number was measured by absolute real-time RT-PCR. (young: n=18 oocytes, control: n=11, PTS: n=13 oocytes). The bars represent the mean ± SE. Different letter (**A**, **B**) indicate significant differences (*p* < 0.05) between the same symbols.

To assess the effects of pterostilbene on the age-related decline of mitochondrial functions, mitochondrial membrane potential in oocytes was determined by measuring fluorescence signal intensity by MitoTracker™ dye staining. As shown in Figures 4A, 4B, the intensity in oocytes derived from aged mice decreased with age. However, the mitochondrial membrane potential in oocytes from mice with long-term pterostilbene ingestion (22 weeks) was significantly higher compared to aged controls. Furthermore, ATP content in oocytes from aged mice with pterostilbene ingestion was significantly higher than in those without pterostilbene ingestion (Figure 4C). While the copy number of mitochondrial DNA in oocytes decreased with animal aged, pterostilbene ingestion did not alter these copy numbers (Figure 4D).

### Pterostilbene did not affect decidualization in endometrial stromal cells

Because recent reports suggest that resveratrol treatment inhibits uterine endometrial decidualization using primary cultures of human endometrial stromal cells and negatively impacts embryo implantation [20, 21], the potential of pterostilbene to suppress endometrial decidualization was investigated in a human endometrial stromal cell line. Given the negative effects of resveratrol on endometrial decidualization assessed in human primary cells and the challenges associated with obtaining human clinical samples, the telomerase-immortalized cell line (THESC) [22] was used in this study.

**Figure 5:**
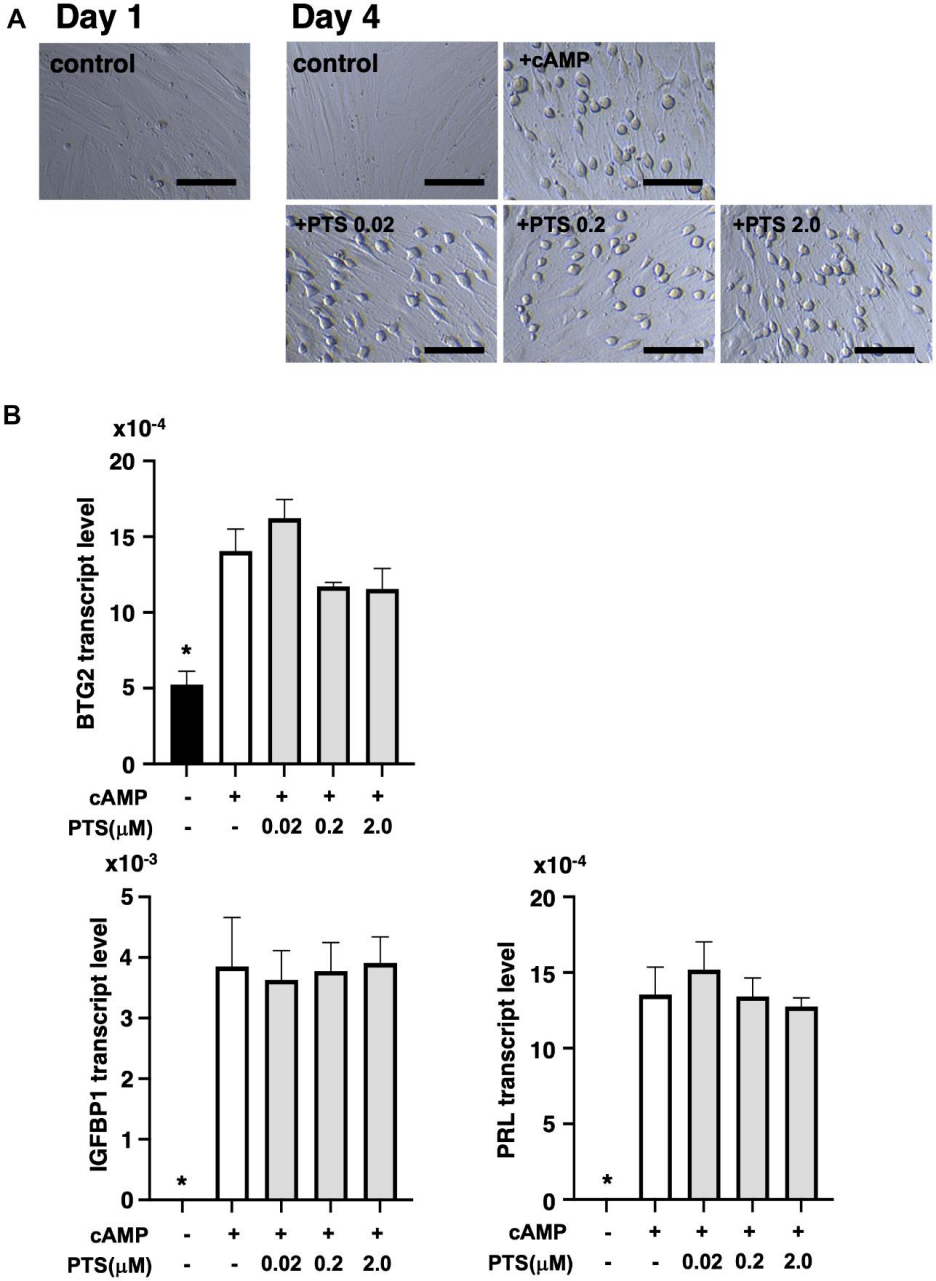
**Effects of pterostilbene ingestion on morphological and genetic markers of uterine endometrial decidualization in the human endometrial stromal cell line (THESC).** The THESC cells were cultured and then *in vitro* decidualization was induced using 8-bromo-cAMP (cAMP) without or with different doses of pterostilbene (PTS) for four days. The untreated group served as a control. (**A**) Morphological changes in THESC before and after decidualization induction. The cells displayed a cobblestone-like morphology following decidualization. Scale bars: 50 mm. (**B**) Changes in genetic markers for endometrial decidualization. The transcript levels of BTG2, IGFBP1, and PRL genes in THESC cells were measured by real-time RT-PCR following decidualization induction. The bars represent mean ± SE. ^*^, *p* < 0.05, significant differences compared to the group treated with cAMP without PTS.

At five days after decidualization induction by 8-bromo-cAMP treatment, THESC cells displayed a cobblestone-like morphology, which is a typical morphological change in decidualized cells (Figure 5A). Similar morphological changes were observed in the groups treated with 0.02-2.0 μM pterostilbene under 8-bromo-cAMP treatment for decidualization induction (Figure 5A). The changes in genetic markers for endometrial decidualization were further analyzed after pterostilbene treatment (Figure 5B). The expression of BTG2, an indicator of the initiation of differentiation into decidualized cells [23], was significantly increased by decidualization induction but remained unchanged across the different doses of pterostilbene treatment. Additionally, the expression of widely used decidualization markers, IGFBP1 and PRL [24], was not affected by pterostilbene treatment (Figure 5B).

## DISCUSSION

The present study demonstrated the anti-aging activity of pterostilbene on age-related infertility using *in vivo* animal models. Pterostilbene restored oocyte quality in aged mice and prevented age-related deterioration of oocyte quality in aged mice without any adverse effects on the animals or their offspring.

The *in vivo* study was designed to assess the effects of pterostilbene on restoring the declined quality of oocytes in aged mice. Mice at 46 weeks of age, which had exhibited a decline in oocyte quality [1, 2, 8], were administered pterostilbene for one week. To investigate the effects of pterostilbene on preventing age-related decline in oocyte quality, adult mice with normal reproductive function were administered pterostilbene in middle age for the long term. While oocyte quality is often assessed based on morphological criteria, its functional definition encompasses reproductive outcomes such as embryonic developmental potential and implantation success, and postnatal health [25, 26]. Notably, previous studies have explicitly linked implantation and live offspring rates to oocyte quality [27–29]. Using implantation and live offspring rates as indicators of oocyte quality, we found that the implantation rate in the short-term pterostilbene administration group was significantly higher than in the control group; however, the increase in live offspring did not reach statistical significance. These results suggest that pterostilbene was unable to fully restore the diminished oocyte quality. The lack of a significant increase in live offspring rate may be attributable to the irreversible age-related declines in oocyte quality, such as chromosomal abnormalities [30]. Further studies are required to clarify this issue. In addition, since long-term pterostilbene ingest significantly increased both implantation and live offspring rates compared to the control group, these findings suggest that pterostilbene may have the potential to prevent age-related decline in oocyte quality. Regarding other indicators of oocyte quality [31], the fertilization and blastocyst formation rates could not be evaluated because aging did not affect these parameters in the animals used for this study.

The present study further demonstrated the beneficial effect of pterostilbene on increasing the number of ovulated oocytes in mice with long-term pterostilbene ingestion, suggesting its preventive effect on the age-related decrease in oocytes numbers. However, no increase in ovulated oocytes was observed in the short-term ingestion group. This could be due to the progressive decline in the number of residual oocytes in the ovary with aging [1, 32], making it impossible to restore the number of ovulated oocytes in aged mice that already have a limited oocyte pool. In this study, the mechanisms underlying the ability of pterostilbene to prevent age-related decline in the number of ovulated oocytes have not been fully elucidated. A possible mechanism of pterostilbene treatment for inhibiting age-related decline in ovulated oocytes includes suppressing follicular atresia during follicle development [33, 34] by inhibiting ovarian apoptosis associated with aging, which has been demonstrated in aging chickens [35].

In this study, prolonged estrous cycles were observed in aged mice; however, no effects of pterostilbene ingestion on the estrous cycle were detected in either the short-term and long-term ingestion groups. One factor contributing to the prolonged estrous cycle observed in aged mice is the age-related decrease in the number of ovarian follicles. In aged mice, the reduction in maturing follicles results in insufficient estradiol secretion and a delayed preovulatory rise in estradiol, leading to prolongation and irregularity of the estrous cycle [36]. In our study, there was no difference in the number of ovulations between the short-term pterostilbene ingestion group and the control group. However, the long-term ingestion group showed a significantly higher number of ovulations compared to controls. These results suggest that short-term pterostilbene ingestion does not promote follicular development, whereas long-term ingestion may help prevent the age-related decline in follicle numbers and support the maintenance of follicle counts. Nevertheless, this protective effect is modest, as the number of ovulated oocytes does not return to the levels observed in young mice, nor does the estrous cycle fully recover to that of young mice.

In oocytes, both the number of mitochondria in the cytoplasm and their activity decrease with age [37, 38], which is one of the causes of declined oocyte quality [39, 40]. It is well established that restoring mitochondrial function can improve oocyte quality and thereby enhance fertility outcomes [41]. During aging, accumulated reactive oxygen species act as oxidative stressors [42] and decrease ATP levels and cell membrane potential in oocytes, which in turn leads to a reduction in oocyte quality [43]. This study demonstrated that long-term ingestion of pterostilbene prevented age-related decline in mitochondrial activity but not mitochondrial number. Similar effects have recently been reported with other dietary supplements (coenzyme Q10 and nicotinamide mononucleotide), which restored mitochondrial activity but not mitochondrial number in aged mouse oocytes [40, 44].

To compare the anti-aging efficacy of pterostilbene with resveratrol on age-related infertility, animals were ingested the same daily dose (6 g/body of 0.04% pterostilbene or resveratrol-containing diet) and treated according to the same study design [15]. Although the implantation rate in aged mice was not restored by short-term use of resveratrol [15], short-term ingestion of pterostilbene ameliorated the implantation rate. These data suggest a superior effect of pterostilbene in restoring oocyte quality in aged animals compared to resveratrol. Additionally, long-term use of resveratrol showed no effect on the number of ovulated oocytes [15], whereas long-term ingestion of pterostilbene increased this number. Therefore, pterostilbene demonstrates a potentially superior preventive effect against age-related decline in ovarian oocyte numbers compared to resveratrol. These advantages of pterostilbene may be attributed to its enhanced pharmacological properties, such as increased lipophilicity, which improves its membrane permeability, bioavailability, and biological potency compared to resveratrol [45]. Furthermore, consistent with previous reports [16, 17], the serum levels of pterostilbene were substantially higher than those of resveratrol shown in our previous study [15], indicating that pterostilbene can maintain stable high concentrations *in vivo*. A previous report supported the advantage of pterostilbene for biological activity, demonstrating that pterostilbene improved cognitive function in a mouse model of dementia, which was not achieved with resveratrol treatment [46]. Of note, we did not perform a direct comparison between pterostilbene and resveratrol in our experimental design. This lack of a direct comparison limits the conclusions that can be drawn regarding their relative efficacy.

A recent study revealed a decrease in clinical pregnancy rate and an increased risk of miscarriage in patients undergoing IVF-ET treatment with resveratrol [20]. Additionally, the decidualization of primary cultured human endometrial stromal cells was inhibited by treatment with 100 μM resveratrol [21]. Therefore, to address the safety of pterostilbene for infertility treatments, we conducted an *in vitro* analysis to determine whether pterostilbene inhibits decidualization. Based on the serum pterostilbene levels obtained from this study in mice (mean 0.02 μM) and clinical studies demonstrating anti-inflammatory effects in healthy humans (0.10-0.15 μM) [47], we tested pterostilbene concentrations ranging from 0.02-2.00 μM. Our findings from cell cultures of THESC indicate that pterostilbene does not inhibit THESC decidualization within this range. However, when cells were treated with an extremely high dose (100 μM) of pterostilbene, the same concentration used in the previous study for resveratrol [21], it was found to be cytotoxic, resulting in cellular degeneration (Supplementary Figure 2). Thus, although different cell types were used in the evaluation, our findings suggest that pterostilbene does not inhibit the decidualization of endometrial stromal cells, at least within the range of concentrations showing beneficial anti-aging activities on age-related infertility in mice.

In our previous study on resveratrol treatment in aging mice, we observed a positive correlation between serum resveratrol concentration and the expression levels of Sirtuin1, 3, 4, 5, and 7 in the ovary [15]. However, no such correlation was found between the serum pterostilbene concentration and the expression of Sirtuin family genes in the ovary (Supplementary Figure 1). These results suggest that pterostilbene and resveratrol may have distinct mechanisms of action on the ovary and oocytes. Previous studies have confirmed that pterostilbene ingestion has anti-aging effects on the brains of mouse models with Alzheimer's disease. However, pterostilbene ingestion did not result in increased Sirt1 expression or downstream markers of Sirt1 activation in the mouse hippocampus, as reported in a previous study [46]. This similarity with the present study suggests that increased Sirt1 expression may not be necessary for the anti-aging effects of pterostilbene. In contrast, studies have reported that pterostilbene can ameliorate liver injury in sepsis model mice and reduce cardiotoxicity in doxorubicin-treated mice by upregulating Sirt1 gene expression [48, 49]. A recent study using small white follicle cultures from chicken ovaries has demonstrated that pterostilbene pretreatment reduced oxidative stress induced by D-galactose and ameliorated apoptosis by upregulation SIRT1 [35]. Therefore, the impact of pterostilbene on sirt1 expression may be tissue- and species-specific.

Previous studies have shown that oxidative stress accumulates in ovarian granulosa cells with age, inducing mitochondrial dysfunction and DNA damage, which results in reduced oocyte quality and abnormal ovarian function [50, 51]. Additionally, exposure to oxidative stress during *in vitro* culture has been linked to a decline in blastocyst formation rate and cell number [52]. In mouse preimplantation embryos, pterostilbene treatment has been shown to protect embryos from H2O2-induced apoptosis through the nuclear factor erythroid 2 like 2 (NFE2L2) pathway by increasing the expression of antioxidant superoxide dismutase (SOD) and regulating the expression of apoptotic factors, B-cell/CLL lymphoma 2 (Bcl-2) and Bcl-2-associated X protein (Bax) [53]. However, we could not find a correlation between the mRNA expression levels of SOD, Bcl-2, and BAX in the ovary and serum pterostilbene concentration (Supplementary Figure 3). This discrepancy may be due to differences in cell types, and further studies are needed to analyze the specific signals that pterostilbene exerts on ovarian cells.

In our study, we found that pterostilbene intake restored mitochondrial activity in oocytes. However, our results suggest that mechanisms other than the SIRT1 and superoxide dismutase pathways may be involved. Several alternative pathways have been reported in the literature. For example, pterostilbene has been shown to activate the heme oxygenase-1 (HO-1) signaling pathway, which suppresses mitochondrial oxidative damage in models of cerebral ischemia-reperfusion injury [54]. In mice, pterostilbene protects cardiomyocytes from oxidative stress and mitochondrial damage induced by acute doxorubicin exposure by activating the adenosine monophosphate-activated protein kinase (AMPK) cascade, leading to upregulation and deacetylation of peroxisome proliferator-activated receptor gamma coactivator 1-alpha (PGC1α) [55]. Studies using human and mouse cell lines have also demonstrated that pterostilbene activates nuclear factor erythroid 2-related factor 2 (Nrf2), suppresses the decrease in mitochondrial membrane potential and the release of cytochrome c, and inhibits the mitochondria-dependent apoptotic pathway [56]. Furthermore, in human glioma cells, pterostilbene has been reported to activate the extracellular signal-regulated kinase 1/2 (ERK1/2) and c-Jun N-terminal kinase (JNK) signaling pathways, induce intrinsic mitochondria-mediated apoptosis, and suppress cell migration and invasion [57].

Understanding the optimal timing for starting supplements to achieve benefits is crucial for clinical application. Continuous supplementation from a young age is challenging, so determining the appropriate time to begin supplementation is essential. In this animal study, the 1-week and 6-week ingestion groups started taking pterostilbene at 46 and 41 weeks respectively, which corresponds to human females beginning at approximately 45 and 40 years of age [58]. Although the short-term (one week) ingestion group did not show a statistically significant difference in live offspring rate, the mean value in this group was 2.7 times higher than that of the aged control group. More importantly, in the short-term ingestion group demonstrated a significant restoration in implantation rate - an important parameter for evaluating oocyte quality - compared to the aged control group. These findings suggest that even short-term pterostilbene ingestion may have the potential to restore oocyte quality. Extrapolating these results to humans, it is expected that oocyte quality in women over 40 years of age could be restored with one or more cycles of pterostilbene ingestion. Meanwhile, to prevent a decline in oocyte quality, restorations in implantation, live offspring, and abortion rates were observed after 22 weeks of pterostilbene ingestion starting at 25 weeks of age, corresponding to women around 30 years of age [58]. Therefore, these results suggest that commencing pterostilbene ingestion before the age of 36, when ovarian function is known to start declining [59], could prevent a decrease in oocyte quality and enhance future fertility. Nevertheless, we acknowledge that the absence of statistical significance in the live offspring rate for the short-term group limits the strength of conclusions regarding its clinical applicability, particularly for women in their 40s. Further investigation, including well-designed human clinical trials, is warranted to clarify the feasibility and efficacy of short-term pterostilbene administration in clinical settings.

This study demonstrated that pterostilbene ingestion restored oocyte quality in aged mice and prevented the age-related decline in both oocyte quality and quantity. While there is currently no established treatment to improve the age-related decline in oocyte quality, which is a major cause of age-related infertility [60], pterostilbene supplementation may provide a solution to this issue. Furthermore, the number of oocytes decreases with age, leading to increased anovulatory cycles [61] and reduced numbers of oocytes retrieved during IVF treatment [62]. Pterostilbene ingestion may potentially preserve ovarian reserve during aging. These effects are expected to contribute to restoring fertility in patients with aging-related infertility and preserving fertility in women planning future childbearing. To date, there have been no reports of adverse events in humans [47, 63], mice [64, 65], or rats [66] at pterostilbene doses similar to or higher than those used in this study. The ingestion of pterostilbene did not affect the body weight of the animals, and no significant abnormalities were detected during rearing. Furthermore, the normality of the offspring from mice ingested with pterostilbene up to the third generation was confirmed. Therefore, these data indicate the safety of pterostilbene ingestion. To clarify the effects of pterostilbene on improving oocyte quality in humans, we are currently conducting a placebo-controlled, randomized, double-blind clinical trial (jRCTs031220638) in older women to investigate the anti-aging effects of pterostilbene on oocyte quality. The results will provide clear evidence of the efficacy of pterostilbene treatment for age-related infertility.

## MATERIALS AND METHODS

### Animals

Male and female ICR mice were purchased from CLEA Japan, Inc. (Tokyo, Japan). The mice were housed at a temperature of 22° C and humidity of 55% with a 12-hour light/12-hour dark cycle and were allowed ad libitum access to food and water. Estrous cycles were monitored every two days by examining vaginal epithelial cell smears. The vaginal smear method used in this study is commonly employed in other research and is considered to have minimal, if any, effect on the estrous cycle in mice [67, 68]. Animals were handled and housed following the procedures specified by the Department of Animal Experiments at the International University of Health and Welfare School of Medicine. All animal experiments were approved by the Animal Care and Use Committee at the International University of Health and Welfare School of Medicine (approval number: 19002NA).

### Protocol for pterostilbene ingestion

Eighty female ICR mice at 25 weeks of age were randomly divided into four groups (n=20 per group) and housed five mice per cage. These mice were fed a diet (6 g per day) containing 0.04% (w/w) pterostilbene (SABINSA JAPAN Co., Ltd., Tokyo, Japan) (Pterostilbene diet: PD), as previously described [69, 70], or a control diet (CD). The four groups were classified based on the duration of pterostilbene feeding (0, 1, 6, and 22 weeks): 1) control group: fed CD throughout the breeding period, 2) 1-week PD group: fed CD until 46 weeks of age, and then fed PD for one week, 3) 6-week PD group: fed CD until 41 weeks of age, and then fed PD from six weeks, and 4) 22-week group: fed PD throughout the breeding period (Figure 1A). As most ICR mice stopped ovulation at about 50 weeks of age in the preliminary survey (data not shown), we used mice at 47 weeks of age with ovulatory competence to analyze the anti-aging activity of pterostilbene on fertility. Some animals died during the long-term breeding period and did not reach 47 weeks of age (1-week PD group: n=1; 22-week PD group: n=2).

Pterostilbene ingestion began at 25 weeks of age and continued for 22 weeks, consistent with our previous study assessing the prevention of oocyte quality decline during aging [14]. Shorter treatment periods of six weeks (twice the time required for mouse primordial follicles to ovulate) and one week were used to assess the restoration of declined oocyte quality of aged mice.

### Mouse physical examinations

To evaluate estrous cycles, vaginal epithelial cell smears were examined for all mice every two days. Body weights were measured at the start (25 weeks of age) and the end (47 weeks of age) of experiments. By the end of the study, the number of animals in the 1-week and 22-week pterostilbene ingestion groups had decreased to 19 and 18, respectively.

### IVF-ET

The estrous cycle was monitored daily in all mice when they reached 47 weeks of age. Mice at the proestrous stage received an intraperitoneal injection of 10 IU human chorionic gonadotropin (hCG; ASKA Pharmaceutical, Tokyo, Japan). One mouse in each of the one-week and six-week pterostilbene ingestion groups remained at the diestrous stage (constant diestrous) and could not be used for experiments requiring ovulation induction. ICR mice at 6 weeks of age served as young controls. At 15 hours post-hCG administration, mice were euthanized, and cumulus-oocyte complexes (COCs) were collected from the oviductal ampulla. COCs were then placed in 100 μl of TYH medium (LSI Medience Corporation, Tokyo, Japan) with sperm (3 x 10^5^ /ml). The sperm were collected from male ICR mice at 10 to 12 weeks of age and incubated in TYH medium for 10 minutes at 37° C under 5% CO_2_/95% air to complete capacitation.

At 5 to 6 hours post-incubation with sperm for fertilization, the inseminated oocytes were collected from COCs and transferred to a 30 μl drop of KSOM medium (Merck Millipore Corporation, Tokyo, Japan) covered with mineral oil (Irvine Scientific Sales Company Inc., Saitama, Japan). The oocytes were then incubated at 37° C for 24 hours. Two-cell stage embryos were selected as fertilized embryos and cultured for an additional 72 hours to form blastocysts. The fertilization rate was calculated as the number of two-cell stage embryos divided by the number of ovulated oocytes, while the blastocyst formation rate was determined as the number of blastocysts divided by the number of two-cell stage embryos.

After culture, the blastocysts derived from individual animals were transferred to the uteri of pseudo-pregnant ICR recipient mice aged 6 to 10 weeks. The pseudo-pregnant ICR recipient mice did not receive pterostilbene at any time, thereby eliminating uterine receptivity or pregnancy maintenance in the recipients as confounding factors. Cesarean sections were performed 16 days after embryo transfer, and the numbers of implantation sites and live fetuses were counted. The implantation rate was calculated as the number of implanted blastocysts divided by the number of transferred blastocysts. The live offspring rate was determined as the number of live fetuses divided by the number of transferred blastocysts, while the abortion rate was calculated as 1 minus the live offspring rate. The gross morphology of placentas and fetuses was evaluated during Cesarean section. The offspring were nursed by foster mothers and mated at 8 weeks of age to assess their fertility.

### Real-time RT-PCR for measurement of Sirtuin family gene expression in ovary

Ovaries were obtained from mice after COC collection, and six ovaries from each group were randomly selected for real-time RT-PCR analysis. Total RNA was extracted using a RNeasy Mini kit (QIAGEN Sciences, Valencia, CA, USA), and cDNA was synthesized using PrimeScript™ RT Master Mix (Takara, Tokyo, Japan) according to the manufacturer’s protocol. Quantitative real-time RT-PCR was performed using Power SYBR® Green PCR Master Mix (Thermo Fisher Scientific, Waltham, MA, USA) on a SmartCycler (Takara) as previously described [71, 72]. The protocol for real-time PCR was as follows: 15 minutes at 95° C, followed by 45 cycles of 15 seconds at 95° C and 60 seconds at 60° C. The primers used are shown in Table 1. Data were normalized based on histone H2a transcript levels. Triplicate measurements were performed in each sample and the mean values were used for data analyses.

**Table 1:** List of primers for real-time RT-PCR

**Gene**	**Forward primer**	**Reverse primer**
**Sirtuin1**	CCTTGGAGACTGCGATGTTA	GTGTTGGTGGCAACTCTGAT
**Sirtuin2**	GCAGTGTCAGAGCGTGGTAA	CTAGTGGTGCCTTGCTGATG
**Sirtuin3**	CTGACTTCGCTTTGGCAGAT	GTCCACCAGCCTTTCCACAC
**Sirtuin4**	GCTTGCCTGAAGCTGGATT	GATCTTGAGCAGCGGAACTC
**Sirtuin5**	AGCCAGAGACTCAAGACGCCA	AGGGCGAGCTCTCTGTCCACC
**Sirtuin6**	TCGGGCCTGTAGAGGGGAGC	CGGCGCTTAGTGGCAAGGGG
**Sirtuin7**	GGCACTTGGTTGTCTACACG	GTGATGCTCATGTGGGTGAG
**Histon-H2a**	ACGAGGAGCTCAACAAGCTG	TATGGTGGCTCTCCGTCTTC
**h-BTG2**	ACGGGAAGGGAACCGACAT	CAGTGGTGTTTGTAGTGCTCTG
**h-IGFBP1**	CTATGATGGCTCGAAGGCTC	TTCTTGTTGCAGTTTGGCAG
**h-PRL**	CATCAACAGCTGCCACACTT	CGTTTGGTTTGCTCCTCAAT
**h-SIRT1**	TCTAACTGGAGCTGGGGTG	AAGTCTACAGCAAGGCGAGC
**h-GAPDH**	TTAAAAGCAGCCCTGGTGAC	CTCTGCTCCTCCTGTTCGAC

### Analysis of mitochondrial membrane potential, ATP content, and mitochondrial DNA copy number in oocytes

Additional animals at 25 weeks of age were fed with or without pterostilbene for 22 weeks for mitochondrial analysis (n=20 per group). Oocytes were collected from ovulated COCs by removing cumulus cells through mechanical pipetting after 1-2 minutes of treatment with 300 μg/ml hyaluronidase (Merck). For young controls, oocytes were obtained from ICR mice at 6 weeks of age using the same procedure (n=5).

The oocytes were incubated with MitoTracker™ Orange (Thermo Fisher Scientific) followed by nuclear staining using Hoechst 33342 dye (Thermo Fisher Scientific) according to the manufacturer’s protocol (young control: n=27, aged control: n=29, pterostilbene ingestion: n=19). After incubation, the mitochondrial membrane potential was visualized using a confocal laser scanning microscope (ZEISS, Oberkochen, Germany), and the fluorescence intensity was quantified using ZEN imaging software (ZEISS).

The ATP content in MII oocytes was determined using an ATP-Glo^™^ Bioluminometric Cell Viability Assay Kit (Biotium, San Francisco, CA, USA) according to the manufacturer’s protocol (n=10 per group). Each individual oocyte was lysed, and its luminescence was measured immediately using a luminometer (Roche, Basel, Switzerland).

Mitochondrial DNA copy number was also measured by real-time PCR according to a previously published method with modifications [73] (n=8 per group). Briefly, an MII oocyte was placed in Tyrode solution (Merck) to remove the zona pellucida and first polar body. Each oocyte was then loaded into a PCR tube with 6 μl of lysis buffer (20 mM Tris, 0.4 mg/ml proteinase K, 0.9% Nonidet-40, and 0.9% Tween 20) and incubated for two hours at 55° C. Proteinase K was subsequently inactivated by heating the samples for 10 minutes at 95° C, after which they were subjected directly to PCR analysis.

Quantitative real-time PCR was performed using Power SYBR® Green PCR Master Mix with previously established probe (B6) and primers (B6-forward and reverse) designed for specific amplification of mouse mtDNA [73]. To generate the standard curve for quantification, PCR products amplified with B6 forward and reverse primers were ligated into a T-vector. Twenty-five-, 50-, and 100-fold serial dilutions of purified plasmid standard DNA were used to generate the standard curve. Triplicate measurements were performed for each sample, and the mean values were used for data analyses.

### Measurement of serum pterostilbene levels

Mouse blood was collected via cardiac puncture using a 1 ml syringe with a 25G needle immediately after euthanasia for COC collection. Serum was then separated by centrifugation the blood at 900 g for 10 minutes at room temperature and collecting the supernatant. As more than 500 μl of serum was required to measure resveratrol levels using HPLC-MS/MS (Nexera X2 system controlled by CBM-20A, Shimadzu Corporation, Kyoto, Japan; triple quadrupole AB-Sciex model API 5000 mass spectrometer, AB-Sciex, Ontario, Canada), animals from which sufficient amounts of serum could not be recovered were excluded from the study.

To prepare the sample for HPLC-MS/MS analysis, 10 μl of internal standard solution (25 ng/ml Trazamide, Fujifilm Wako Pure Chemical Corporation, Osaka, Japan) and 10 μl borate buffer (pH 9.18) were added to 50 μl of each serum sample. The mixture was vortexed for 10 seconds. Then, 800 μl of ethyl acetate was added to the mixture. After 3 minutes of vortexing, the mixture was centrifuged at 4° C for 2 minutes at 5,000 × g. The organic layer was transferred to a glass tube and evaporated to dryness under a nitrogen stream at 40° C. The residue was reconstituted in 50 μl of methanol, vortexed for 30 seconds, and sonicated for one minute to ensure complete dissolution. Subsequently, 150 μl of water was added to the mixture and vortexed for 30 seconds. After centrifugation at 4° C for 3 minutes at 2,000 × g, the supernatant was transferred into the HPLC-MS/MS system for analysis.

The identification and quantification of pterostilbene and its metabolites in serum were conducted by HPLC-MS/MS according to the manufacturer’s protocol. Serum samples were analyzed by HPLC separation using a CAPCELL PAK C18 MG II column (Shiseido, Tokyo, Japan). A 10 mM ammonium acetate solution was used as mobile phase A, and methanol as mobile phase B. Samples were injected into the column maintained at 40° C. Mobile phases A and B were eluted at a flow rate of 0.3 ml/minute with a linear gradient in which the volume ratio changed from 80:20 to 0:100. The gradient elution was performed as follows: 20% B (0–0.5 minutes), 80% B (0.5–6.5 minutes), 100% B (6.51–7.50 minutes), and then 20% B (7.5–10.5 minutes).

The system was equipped with an electrospray ionization source and operated in negative ion mode with multiple reaction monitoring. The tune method was set as follows: collision gas (nitrogen) flow rate of 6 arb, curtain gas (nitrogen) flow rate of 10 arb, nebulizer gas (air) flow rate of 60 arb, desolvation gas (air) flow rate of 60 arb, ion spray voltage of 4.5 kV, entrance potential of 10 V, and collision cell exit potential of 10 V. The monitored ion transitions were m/z 227-143 (Res) and m/z 310-170 (trazamide).

### Cell culture and decidualization

The THESC cells were obtained from the American Type Culture Collection (CRL-4003; ATCC, Manassas, VA, USA) and cultured according to the supplier's instructions. Cells were maintained in phenol red-free DMEM/F12 medium (Thermo Fisher Scientific) supplemented with 10% fetal bovine serum (FBS), 100 units/ml penicillin, 0.1 mg/ml streptomycin, and 1% insulin-transferrin-selenium (ITS) + Premix (BD Biosciences, Franklin Lakes, NJ, USA). *In vitro* decidualization of THESC was induced as described previously, with some modifications [74]. Briefly, cells were seeded to confluence in 96-well plates and stimulated the following day with 1.0 mM 8-bromo-cAMP (Sigma-Aldrich, St. Louis, MO, USA) in phenol red-free DMEM/F12 medium containing 0.1% FBS, 100 units/ml penicillin, 0.1 mg/ml streptomycin and 1% ITS+ Premix, without or with 0.02, 0.2, and 2.0 μM pterostilbene for four days to induce decidualization. The medium was changed every 48 hours. The non-treated group served as a control. In some experiments, THESC cells were cultured in the phenol red-free DMEM/F12 medium with 1.0 mM 8-bromo-cAMP and either 50 or 100 μM pterostilbene for 24 hours.

### Real-time RT-PCR for measurement of SIRT1, BTG2, IGFBP1, and PRL gene expression in THESC cells during decidualization

Total RNA was extracted by direct lysis in the culture plates using the RNeasy Micro kit (QIAGEN) according to the manufacturer's instructions. Real-time RT-PCR was performed as described above. Data were normalized based on glyceraldehyde-3-phosphate dehydrogenase (GAPDH) transcript levels. The primers used are listed in Table 1.

### Statistical analysis

The results were expressed as the mean ± standard error (SE). Intergroup comparisons were performed using one-way ANOVA, followed by Dunnett’s test for multiple comparisons. The level of significance was set at *p* < 0.05. The correlation between serum pterostilbene levels and Sirtuin mRNA expression levels, implantation rates, and live offspring rates was analyzed using Pearson's correlation coefficient.

A correlation coefficient (r) value closer to 1 indicates a stronger positive correlation, while an r value closer to -1 indicates a stronger negative correlation. An r value near 0 suggests little or no correlation. A r value above 0.3 was considered to indicate a significant correlation.

## Supplementary Material

Supplementary Figures

## References

[1] Broekmans FJ, Soules MR, Fauser BC. Ovarian aging: mechanisms and clinical consequences. Endocr Rev. 2009; 30:465–93. 10.1210/er.2009-0006 19589949

[2] Tatone C, Amicarelli F, Carbone MC, Monteleone P, Caserta D, Marci R, Artini PG, Piomboni P, Focarelli R. Cellular and molecular aspects of ovarian follicle ageing. Hum Reprod Update. 2008; 14:131–42. 10.1093/humupd/dmm048 18239135

[3] Yan J, Wu K, Tang R, Ding L, Chen ZJ. Effect of maternal age on the outcomes of *in vitro* fertilization and embryo transfer (IVF-ET). Sci China Life Sci. 2012; 55:694–8. 10.1007/s11427-012-4357-0 22932885

[4] ESHRE Capri Workshop Group. Genetic aspects of female reproduction. Hum Reprod Update. 2008; 14:293–307. 10.1093/humupd/dmn009 18385259

[5] Schmidt L, Sobotka T, Bentzen JG, Nyboe Andersen A, and ESHRE Reproduction and Society Task Force. Demographic and medical consequences of the postponement of parenthood. Hum Reprod Update. 2012; 18:29–43. 10.1093/humupd/dmr040 21989171

[6] Almansa-Ordonez A, Bellido R, Vassena R, Barragan M, Zambelli F. Oxidative Stress in Reproduction: A Mitochondrial Perspective. Biology (Basel). 2020; 9:269. 10.3390/biology9090269 32899860 PMC7564700

[7] Fujino Y, Ozaki K, Yamamasu S, Ito F, Matsuoka I, Hayashi E, Nakamura H, Ogita S, Sato E, Inoue M. DNA fragmentation of oocytes in aged mice. Hum Reprod. 1996; 11:1480–3. 10.1093/oxfordjournals.humrep.a019421 8671488

[8] Iwata H, Goto H, Tanaka H, Sakaguchi Y, Kimura K, Kuwayama T, Monji Y. Effect of maternal age on mitochondrial DNA copy number, ATP content and IVF outcome of bovine oocytes. Reprod Fertil Dev. 2011; 23:424–32. 10.1071/RD10133 21426860

[9] Pervaiz S, Holme AL. Resveratrol: its biologic targets and functional activity. Antioxid Redox Signal. 2009; 11:2851–97. 10.1089/ars.2008.2412 19432534

[10] Asensi M, Ortega A, Mena S, Feddi F, Estrela JM. Natural polyphenols in cancer therapy. Crit Rev Clin Lab Sci. 2011; 48:197–216. 10.3109/10408363.2011.631268 22141580

[11] Bharadvaja N, Gautam S, Singh H. Natural polyphenols: a promising bioactive compounds for skin care and cosmetics. Mol Biol Rep. 2023; 50:1817–28. 10.1007/s11033-022-08156-9 36494596

[12] Wang S, Du Q, Meng X, Zhang Y. Natural polyphenols: a potential prevention and treatment strategy for metabolic syndrome. Food Funct. 2022; 13:9734–53. 10.1039/d2fo01552h 36134531

[13] Park SJ, Ahmad F, Philp A, Baar K, Williams T, Luo H, Ke H, Rehmann H, Taussig R, Brown AL, Kim MK, Beaven MA, Burgin AB, et al. Resveratrol ameliorates aging-related metabolic phenotypes by inhibiting cAMP phosphodiesterases. Cell. 2012; 148:421–33. 10.1016/j.cell.2012.01.017 22304913 PMC3431801

[14] Liu M, Yin Y, Ye X, Zeng M, Zhao Q, Keefe DL, Liu L. Resveratrol protects against age-associated infertility in mice. Hum Reprod. 2013; 28:707–17. 10.1093/humrep/des437 23293221

[15] Okamoto N, Sato Y, Kawagoe Y, Shimizu T, Kawamura K. Short-term resveratrol treatment restored the quality of oocytes in aging mice. Aging (Albany NY). 2022; 14:5628–40. 10.18632/aging.204157 35802632 PMC9365568

[16] Asensi M, Medina I, Ortega A, Carretero J, Baño MC, Obrador E, Estrela JM. Inhibition of cancer growth by resveratrol is related to its low bioavailability. Free Radic Biol Med. 2002; 33:387–98. 10.1016/s0891-5849(02)00911-5 12126761

[17] Khushnud T, Mousa SA. Potential role of naturally derived polyphenols and their nanotechnology delivery in cancer. Mol Biotechnol. 2013; 55:78–86. 10.1007/s12033-012-9623-7 23371307

[18] Ferrer P, Asensi M, Segarra R, Ortega A, Benlloch M, Obrador E, Varea MT, Asensio G, Jordá L, Estrela JM. Association between pterostilbene and quercetin inhibits metastatic activity of B16 melanoma. Neoplasia. 2005; 7:37–47. 10.1593/neo.04337 15736313 PMC1490314

[19] Ullah O, Li Z, Ali I, Xu L, Liu H, Shah SZ, Fang N. Pterostilbene alleviates hydrogen peroxide-induced oxidative stress via nuclear factor erythroid 2 like 2 pathway in mouse preimplantation embryos. J Reprod Dev. 2019; 65:73–81. 10.1262/jrd.2018-089 30429414 PMC6379763

[20] Ochiai A, Kuroda K, Ikemoto Y, Ozaki R, Nakagawa K, Nojiri S, Takeda S, Sugiyama R. Influence of resveratrol supplementation on IVF-embryo transfer cycle outcomes. Reprod Biomed Online. 2019; 39:205–10. 10.1016/j.rbmo.2019.03.205 31160243

[21] Ochiai A, Kuroda K, Ozaki R, Ikemoto Y, Murakami K, Muter J, Matsumoto A, Itakura A, Brosens JJ, Takeda S. Resveratrol inhibits decidualization by accelerating downregulation of the CRABP2-RAR pathway in differentiating human endometrial stromal cells. Cell Death Dis. 2019; 10:276. 10.1038/s41419-019-1511-7 30894514 PMC6427032

[22] Krikun G, Mor G, Alvero A, Guller S, Schatz F, Sapi E, Rahman M, Caze R, Qumsiyeh M, Lockwood CJ. A novel immortalized human endometrial stromal cell line with normal progestational response. Endocrinology. 2004; 145:2291–6. 10.1210/en.2003-1606 14726435

[23] Donato LJ, Suh JH, Noy N. Suppression of mammary carcinoma cell growth by retinoic acid: the cell cycle control gene Btg2 is a direct target for retinoic acid receptor signaling. Cancer Res. 2007; 67:609–15. 10.1158/0008-5472.CAN-06-0989 17234770

[24] Yu SL, Lee SI, Park HW, Lee SK, Kim TH, Kang J, Park SR. SIRT1 suppresses *in vitro* decidualization of human endometrial stromal cells through the downregulation of forkhead box O1 expression. Reprod Biol. 2022; 22:100672. 10.1016/j.repbio.2022.100672 35839571

[25] Krisher RL. *In vivo* and *in vitro* environmental effects on mammalian oocyte quality. Annu Rev Anim Biosci. 2013; 1:393–417. 10.1146/annurev-animal-031412-103647 25387025

[26] Lemseffer Y, Terret ME, Campillo C, Labrune E. Methods for Assessing Oocyte Quality: A Review of Literature. Biomedicines. 2022; 10:2184. 10.3390/biomedicines10092184 36140285 PMC9495944

[27] Navot D, Bergh PA, Williams MA, Garrisi GJ, Guzman I, Sandler B, Grunfeld L. Poor oocyte quality rather than implantation failure as a cause of age-related decline in female fertility. Lancet. 1991; 337:1375–7. 10.1016/0140-6736(91)93060-m 1674764

[28] Balaban B, Urman B. Effect of oocyte morphology on embryo development and implantation. Reprod Biomed Online. 2006; 12:608–15. 10.1016/s1472-6483(10)61187-x 16790106

[29] Ozturk S. Selection of competent oocytes by morphological criteria for assisted reproductive technologies. Mol Reprod Dev. 2020; 87:1021–36. 10.1002/mrd.23420 32902927

[30] Lushnikova T, Bouska A, Odvody J, Dupont WD, Eischen CM. Aging mice have increased chromosome instability that is exacerbated by elevated Mdm2 expression. Oncogene. 2011; 30:4622–31. 10.1038/onc.2011.172 21602883 PMC3161162

[31] Coticchio G, Dal Canto M, Mignini Renzini M, Guglielmo MC, Brambillasca F, Turchi D, Novara PV, Fadini R. Oocyte maturation: gamete-somatic cells interactions, meiotic resumption, cytoskeletal dynamics and cytoplasmic reorganization. Hum Reprod Update. 2015; 21:427–54. 10.1093/humupd/dmv011 25744083

[32] Findlay JK, Hutt KJ, Hickey M, Anderson RA. How Is the Number of Primordial Follicles in the Ovarian Reserve Established? Biol Reprod. 2015; 93:111. 10.1095/biolreprod.115.133652 26423124

[33] Dumesic DA, Meldrum DR, Katz-Jaffe MG, Krisher RL, Schoolcraft WB. Oocyte environment: follicular fluid and cumulus cells are critical for oocyte health. Fertil Steril. 2015; 103:303–16. 10.1016/j.fertnstert.2014.11.015 25497448

[34] Yao J, Ma Y, Zhou S, Bao T, Mi Y, Zeng W, Li J, Zhang C. Metformin Prevents Follicular Atresia in Aging Laying Chickens through Activation of PI3K/AKT and Calcium Signaling Pathways. Oxid Med Cell Longev. 2020; 2020:3648040. 10.1155/2020/3648040 33294120 PMC7718058

[35] Wang X, Yuan Q, Xiao Y, Cai X, Yang Z, Zeng W, Mi Y, Zhang C. Pterostilbene, a Resveratrol Derivative, Improves Ovary Function by Upregulating Antioxidant Defenses in the Aging Chickens via Increased SIRT1/Nrf2 Expression. Antioxidants (Basel). 2024; 13:935. 10.3390/antiox13080935 39199181 PMC11351833

[36] Nelson JF, Felicio LS, Randall PK, Sims C, Finch CE. A longitudinal study of estrous cyclicity in aging C57BL/6J mice: I. Cycle frequency, length and vaginal cytology. Biol Reprod. 1982; 27:327–39. 10.1095/biolreprod27.2.327 6889895

[37] Chiang JL, Shukla P, Pagidas K, Ahmed NS, Karri S, Gunn DD, Hurd WW, Singh KK. Mitochondria in Ovarian Aging and Reproductive Longevity. Ageing Res Rev. 2020; 63:101168. 10.1016/j.arr.2020.101168 32896666 PMC9375691

[38] Shoubridge EA, Wai T. Mitochondrial DNA and the mammalian oocyte. Curr Top Dev Biol. 2007; 77:87–111. 10.1016/S0070-2153(06)77004-1 17222701

[39] Fragouli E, Spath K, Alfarawati S, Kaper F, Craig A, Michel CE, Kokocinski F, Cohen J, Munne S, Wells D. Altered levels of mitochondrial DNA are associated with female age, aneuploidy, and provide an independent measure of embryonic implantation potential. PLoS Genet. 2015; 11:e1005241. 10.1371/journal.pgen.1005241 26039092 PMC4454688

[40] Ben-Meir A, Burstein E, Borrego-Alvarez A, Chong J, Wong E, Yavorska T, Naranian T, Chi M, Wang Y, Bentov Y, Alexis J, Meriano J, Sung HK, et al. Coenzyme Q10 restores oocyte mitochondrial function and fertility during reproductive aging. Aging Cell. 2015; 14:887–95. 10.1111/acel.12368 26111777 PMC4568976

[41] Bron AJ, Yokoi N, Gafney E, Tiffany JM. Predicted phenotypes of dry eye: proposed consequences of its natural history. Ocul Surf. 2009; 7:78–92. 10.1016/s1542-0124(12)70299-9 19383277

[42] Kudryavtseva AV, Krasnov GS, Dmitriev AA, Alekseev BY, Kardymon OL, Sadritdinova AF, Fedorova MS, Pokrovsky AV, Melnikova NV, Kaprin AD, Moskalev AA, Snezhkina AV. Mitochondrial dysfunction and oxidative stress in aging and cancer. Oncotarget. 2016; 7:44879–905. 10.18632/oncotarget.9821 27270647 PMC5216692

[43] May-Panloup P, Boucret L, Chao de la Barca JM, Desquiret-Dumas V, Ferré-L’Hotellier V, Morinière C, Descamps P, Procaccio V, Reynier P. Ovarian ageing: the role of mitochondria in oocytes and follicles. Hum Reprod Update. 2016; 22:725–43. 10.1093/humupd/dmw028 27562289

[44] Miao Y, Cui Z, Gao Q, Rui R, Xiong B. Nicotinamide Mononucleotide Supplementation Reverses the Declining Quality of Maternally Aged Oocytes. Cell Rep. 2020; 32:107987. 10.1016/j.celrep.2020.107987 32755581

[45] Retraction: Role of mesenchymal stem cells versus angiotensin converting enzyme inhibitor in kidney repair. Nephrology (Carlton). 2024; 29:239. 10.1111/nep.14278 38341878

[46] Chang J, Rimando A, Pallas M, Camins A, Porquet D, Reeves J, Shukitt-Hale B, Smith MA, Joseph JA, Casadesus G. Low-dose pterostilbene, but not resveratrol, is a potent neuromodulator in aging and Alzheimer’s disease. Neurobiol Aging. 2012; 33:2062–71. 10.1016/j.neurobiolaging.2011.08.015 21982274

[47] Hougee S, Faber J, Sanders A, de Jong RB, van den Berg WB, Garssen J, Hoijer MA, Smit HF. Selective COX-2 inhibition by a Pterocarpus marsupium extract characterized by pterostilbene, and its activity in healthy human volunteers. Planta Med. 2005; 71:387–92. 10.1055/s-2005-864130 15931573

[48] Liu D, Ma Z, Xu L, Zhang X, Qiao S, Yuan J. PGC1α activation by pterostilbene ameliorates acute doxorubicin cardiotoxicity by reducing oxidative stress via enhancing AMPK and SIRT1 cascades. Aging (Albany NY). 2019; 11:10061–73. 10.18632/aging.102418 31733141 PMC6914429

[49] Liu X, Yang X, Han L, Ye F, Liu M, Fan W, Zhang K, Kong Y, Zhang J, Shi L, Chen Y, Zhang X, Lin S. Pterostilbene alleviates polymicrobial sepsis-induced liver injury: Possible role of SIRT1 signaling. Int Immunopharmacol. 2017; 49:50–9. 10.1016/j.intimp.2017.05.022 28550734

[50] Taheri M, Hayati Roudbari N, Amidi F, Parivar K. The protective effect of sulforaphane against oxidative stress in granulosa cells of patients with polycystic ovary syndrome (PCOS) through activation of AMPK/AKT/NRF2 signaling pathway. Reprod Biol. 2021; 21:100563. 10.1016/j.repbio.2021.100563 34678578

[51] Agarwal A, Gupta S, Sharma RK. Role of oxidative stress in female reproduction. Reprod Biol Endocrinol. 2005; 3:28. 10.1186/1477-7827-3-28 16018814 PMC1215514

[52] Duranthon V, Watson AJ, Lonergan P. Preimplantation embryo programming: transcription, epigenetics, and culture environment. Reproduction. 2008; 135:141–50. 10.1530/REP-07-0324 18239045

[53] Chen X, Song QL, Li ZH, Ji R, Wang JY, Cao ML, Mu XF, Zhang Y, Guo DY, Yang J. Pterostilbene ameliorates oxidative damage and ferroptosis in human ovarian granulosa cells by regulating the Nrf2/HO-1 pathway. Arch Biochem Biophys. 2023; 738:109561. 10.1016/j.abb.2023.109561 36898621

[54] Cheng YC, Chen PY, Way TD, Cheng CL, Huang YP, Hsia TC, Chou YC, Peng SF. Pre-Treatment of Pterostilbene Enhances H_2_O_2_-induced Cell Apoptosis Through Caspase-dependent Pathway in Human Keratinocyte Cells. In Vivo. 2021; 35:833–43. 10.21873/invivo.12324 33622876 PMC8045064

[55] Zhou G, Myers R, Li Y, Chen Y, Shen X, Fenyk-Melody J, Wu M, Ventre J, Doebber T, Fujii N, Musi N, Hirshman MF, Goodyear LJ, Moller DE. Role of AMP-activated protein kinase in mechanism of metformin action. J Clin Invest. 2001; 108:1167–74. 10.1172/JCI13505 11602624 PMC209533

[56] Zhou J, Ci X, Ma X, Yu Q, Cui Y, Zhen Y, Li S. Pterostilbene Activates the Nrf2-Dependent Antioxidant Response to Ameliorate Arsenic-Induced Intracellular Damage and Apoptosis in Human Keratinocytes. Front Pharmacol. 2019; 10:497. 10.3389/fphar.2019.00497 31139082 PMC6519314

[57] Gao H, Liu Z, Xu W, Wang Q, Zhang C, Ding Y, Nie W, Lai J, Chen Y, Huang H. Pterostilbene promotes mitochondrial apoptosis and inhibits proliferation in glioma cells. Sci Rep. 2021; 11:6381. 10.1038/s41598-021-85908-w 33737656 PMC7973728

[58] Fox JG. The mouse in biomedical research. (Amsterdam; Boston: Elsevier, AP). 2007.

[59] Ahmed TA, Ahmed SM, El-Gammal Z, Shouman S, Ahmed A, Mansour R, El-Badri N. Oocyte Aging: The Role of Cellular and Environmental Factors and Impact on Female Fertility. Adv Exp Med Biol. 2020; 1247:109–23. 10.1007/5584_2019_456 31802446

[60] Moghadam AR, Moghadam MT, Hemadi M, Saki G. Oocyte quality and aging. JBRA Assist Reprod. 2022; 26:105–22. 10.5935/1518-0557.20210026 34338482 PMC8769179

[61] Burger HG. The menopausal transition. Baillieres Clin Obstet Gynaecol. 1996; 10:347–59. 10.1016/s0950-3552(96)80019-8 8931899

[62] Vollenhoven B, Hunt S. Ovarian ageing and the impact on female fertility. F1000Res. 2018; 7:F1000 Faculty Rev-1835. 10.12688/f1000research.16509.1 30542611 PMC6259486

[63] Riche DM, McEwen CL, Riche KD, Sherman JJ, Wofford MR, Deschamp D, Griswold M. Analysis of safety from a human clinical trial with pterostilbene. J Toxicol. 2013; 2013:463595. 10.1155/2013/463595 23431291 PMC3575612

[64] Ruiz MJ, Fernández M, Picó Y, Mañes J, Asensi M, Carda C, Asensio G, Estrela JM. Dietary administration of high doses of pterostilbene and quercetin to mice is not toxic. J Agric Food Chem. 2009; 57:3180–86. 10.1021/jf803579e 19292443

[65] Tsai HY, Shih YY, Yeh YT, Huang CH, Liao CA, Hu CY, Nagabhushanam K, Ho CT, Chen YK. Pterostilbene and Its Derivative 3'-Hydroxypterostilbene Ameliorated Nonalcoholic Fatty Liver Disease Through Synergistic Modulation of the Gut Microbiota and SIRT1/AMPK Signaling Pathway. J Agric Food Chem. 2022; 70:4966–80. 10.1021/acs.jafc.2c00641 35416649

[66] Sun C, Li Y, Zhang Y, Huang H, Chen H, Chen J, Han L, Chen X, Chen X, Zhang Y. Subacute oral toxicology and toxicokinetics of pterostilbene, a novel Top1/Tdp1 inhibiting anti-tumor reagent. Drug Chem Toxicol. 2023; 46:392–9. 10.1080/01480545.2022.2042014 35253568

[67] Caligioni CS. Assessing reproductive status/stages in mice. Curr Protoc Neurosci. 2009; Appendix 4:Appendix 4I. 10.1002/0471142301.nsa04is48 19575469 PMC2755182

[68] Byers SL, Wiles MV, Dunn SL, Taft RA. Mouse estrous cycle identification tool and images. PLoS One. 2012; 7:e35538. 10.1371/journal.pone.0035538 22514749 PMC3325956

[69] Watanabe K, Shibuya S, Ozawa Y, Izuo N, Shimizu T. Resveratrol Derivative-Rich Melinjo Seed Extract Attenuates Skin Atrophy in Sod1-Deficient Mice. Oxid Med Cell Longev. 2015; 2015:391075. 10.1155/2015/391075 26180586 PMC4477213

[70] Baur JA, Sinclair DA. Therapeutic potential of resveratrol: the *in vivo* evidence. Nat Rev Drug Discov. 2006; 5:493–506. 10.1038/nrd2060 16732220

[71] Kawamura K, Fukuda J, Kumagai J, Shimizu Y, Kodama H, Nakamura A, Tanaka T. Gonadotropin-releasing hormone I analog acts as an antiapoptotic factor in mouse blastocysts. Endocrinology. 2005; 146:4105–16. 10.1210/en.2004-1646 15932933

[72] Kawamura K, Kawamura N, Fukuda J, Kumagai J, Hsueh AJ, Tanaka T. Regulation of preimplantation embryo development by brain-derived neurotrophic factor. Dev Biol. 2007; 311:147–58. 10.1016/j.ydbio.2007.08.026 17880937

[73] Shitara H, Kaneda H, Sato A, Inoue K, Ogura A, Yonekawa H, Hayashi JI. Selective and continuous elimination of mitochondria microinjected into mouse eggs from spermatids, but not from liver cells, occurs throughout embryogenesis. Genetics. 2000; 156:1277–84. 10.1093/genetics/156.3.1277 11063701 PMC1461340

[74] Saleh L, Otti GR, Fiala C, Pollheimer J, Knöfler M. Evaluation of human first trimester decidual and telomerase-transformed endometrial stromal cells as model systems of *in vitro* decidualization. Reprod Biol Endocrinol. 2011; 9:155. 10.1186/1477-7827-9-155 22151839 PMC3267678

