# Supplementary material for: Pterostilbene enhances reproductive outcomes and oocyte quality in aged mice without adverse effects
Source: Aging (Albany NY). 2025 Jul 23;17(7):1762–83. doi: 10.18632/aging.206287 (PMC12339035; doi:10.18632/aging.206287)
Supplement: Supplementary Figures [file aging-17-206287-s001.pdf]

SUPPLEMENTARY FIGURES

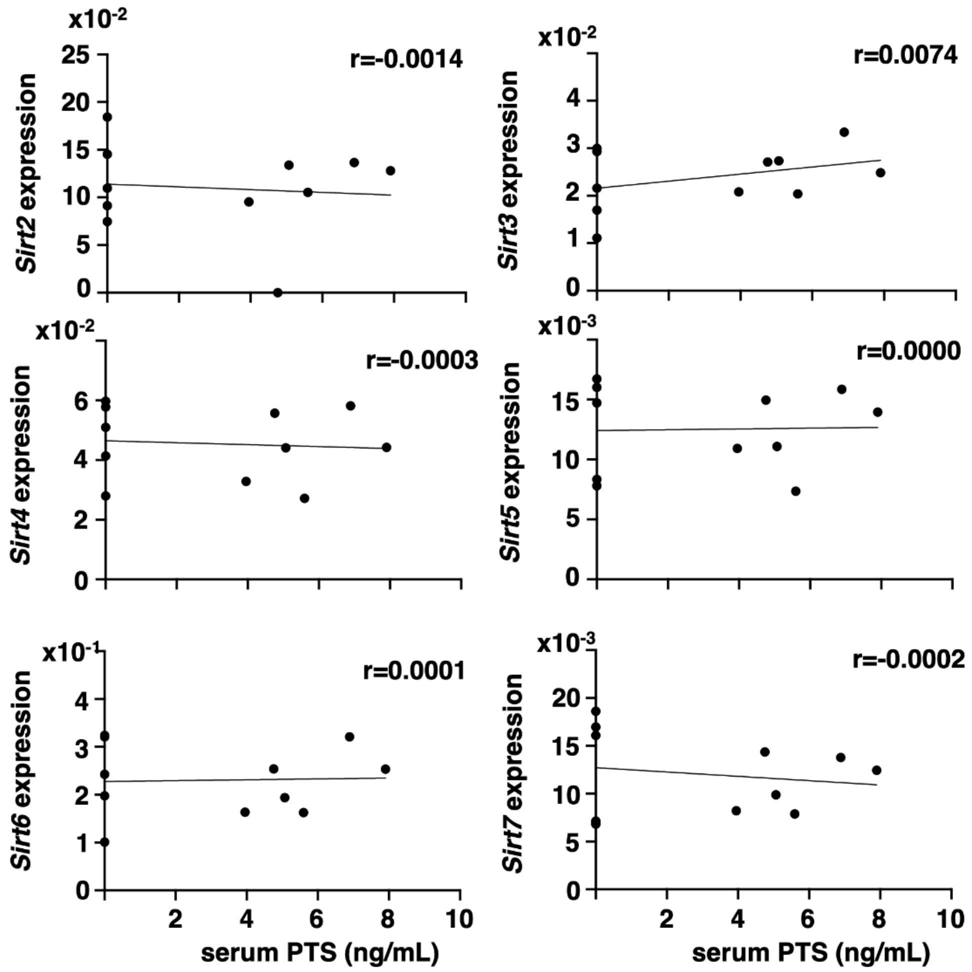

Supplementary Figure 1. Correlation between serum pterostilbene levels and ovarian transcript levels of the Sirtuin family in aged mice. Serum pterostilbene (PTS) levels were quantified in control and 22-week ingestion groups using HPLC-MS/MS, whereas Sirt1 transcript levels were determined by real-time RT-PCR. The primers used are shown in Table 1. No correlation was observed between serum PTS levels and ovarian mRNA expression levels of the Sirtuin family genes (n=11 animals). A correlation coefficient (r) above 0.3 was considered to indicate a significant correlation.

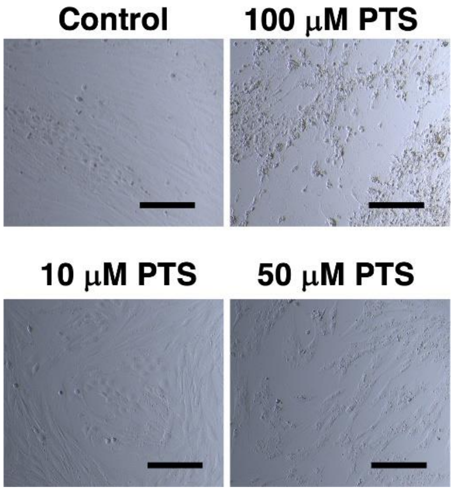

Supplementary Figure 2. Impact of high dose pterostilbene on cell viability in human endometrial stromal cell line (THESC). The THESC cells were cultured with or without a high dose (100 mM) of pterostilbene (PTS). The non-treated group served as a control. Treatment with 100 mM PTS overnight results in cell death. Scale bars: 50 mm.

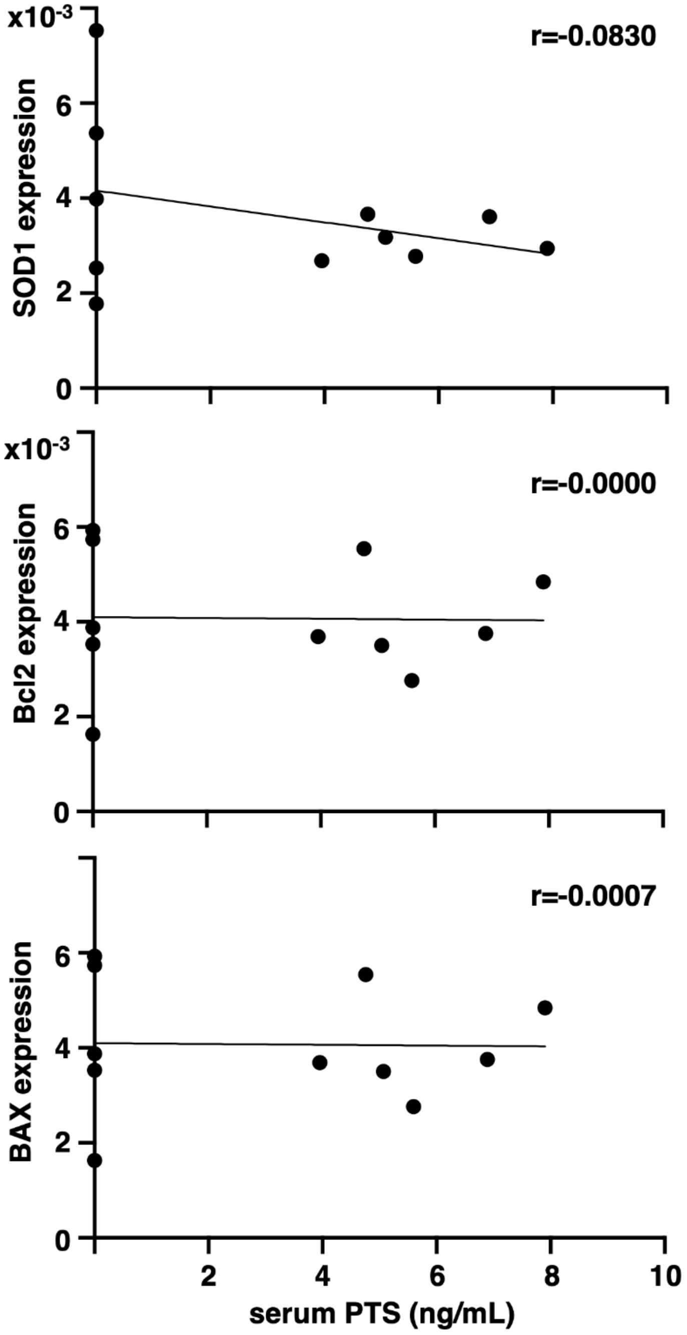

Supplementary Figure 3. Correlation between serum pterostilbene levels and ovarian transcript levels of *Sod1*, *Bcl2*, and *Bax* in aged mice. Serum pterostilbene (PTS) levels were quantified in control and 22-week ingestion groups using HPLC-MS/MS, while *Sod1*, *Bcl2*, and *Bax* transcript levels were determined by real-time RT-PCR. The primers used are shown in Table 1. No correlation was observed between serum PTS levels and ovarian mRNA expression levels of *Sod1*, *Bcl2*, and *Bax* genes (n=11 animals). A correlation coefficient (r) above 0.3 was considered to indicate a significant correlation.
